# Pericardial Tamponade Masquerading as Abdominal Pain Diagnosed by Point-of-care Ultrasonography

**DOI:** 10.5811/cpcem.2017.9.34436

**Published:** 2017-11-03

**Authors:** Usama Khalid, Mark Favot, Farah Ubaid

**Affiliations:** * Emory University, Department of Emergency Medicine, Atlanta, Georgia; † Wayne State University, Department of Emergency Medicine, Detroit, Michigan

## Abstract

An 18-year-old female presented to the emergency department with a complaint of right-sided abdominal pain for one day. An abdominal computed tomography was significant for hepatic congestion and a large pericardial effusion. The patient was found to have early signs of cardiac tamponade on point-of-care ultrasonography. She was taken to the operating room for pericardial window and had immediate resolution of her symptoms. Patient was diagnosed with systemic lupus erythematosus based on laboratory and clinical findings. This case report details the atypical clinical features of our patient and highlights the subtle signs that should indicate the need for point-of-care cardiac ultrasonographic assessment in these patients.

## INTRODUCTION

Systemic lupus erythematosus (SLE) is a multisystem, autoimmune disease that can manifest clinically in a myriad of different presentations. The initial encounter with a previously undiagnosed patient can prove to be a difficult diagnostic dilemma for the emergency physician. Patients can present with any organ system involvement, hence the variety in clinical symptomatology. Although the exact pathophysiology of this disease process is unclear, it is believed to be due to an immune-mediated response to inappropriately recognized self-antigens from a specific organ.^1^

Lupus pericarditis is a well-known entity associated with the underlying disease process. Multiple case reports in the literature document pericarditis with associated pericardial effusion as an initial manifestation of SLE. The classic presentation of pericarditis is well established as sharp chest pain, occasionally with a component of dyspnea and pleurisy. As a pericardial effusion develops, the pressure-volume dynamics cause inadequate ventricular filling due to increasing pericardial volume and thus pressure, resulting in acute right heart failure with subsequent hepatic congestion and jugular venous distension. Additionally, cardiac output also decreases, leading to further hemodynamic compromise.^1^

Hepatic congestion from right heart failure occurs due to elevated filling pressure within the right ventricle (RV) or, in the case of pericardial tamponade, when pericardial pressure exceeds RV diastolic pressure. Subacute or acute processes result in stretching of the liver capsule, leading to right-sided abdominal discomfort. Depending on the chronicity of this process, patients may develop congestion of the portal system or even ascites; however, most patients with pericarditis present with the aforementioned signs and symptoms before any of these complications occur.^2^

Lupus pericarditis is classically treated with anti-inflammatory medications along with immunomodulation. In the case of secondary pericardial effusions, pericardiocentesis or more definitive pericardial window may be needed. This report will detail the case of a patient presenting with abdominal discomfort from hepatic congestion as an initial manifestation of pericardial tamponade from undiagnosed lupus.

## CASE REPORT

An 18-year-old African-American female presented to the emergency department (ED) complaining of abdominal pain in the right upper and lower quadrant. The pain had awoken the patient from sleep a few hours prior to arrival. She also complained of mild palpitations, which were non-exertional in nature. She denied any fevers or chills or any other associated symptoms except for decreased appetite over the previous day. The patient denied any chest pain or dyspnea. She had a past history of hypertension and hyperlipidemia but denied a formal diagnosis. She was not on any anti-hypertensives. On arrival, the patient had an elevated blood pressure of 175/104 mmHg and tachycardia of 120 beats per minute. An electrocardiogram (ECG) was performed upon arrival in light of the tachycardia and was essentially unremarkable except for sinus tachycardia. The patient had been seen two months prior for gastritis and was noted to have an elevated creatinine at that time, which had been attributed to her chronic hypertension. On physical examination, the patient exhibited right upper and lower quadrant tenderness to palpation with an unremarkable pelvic exam. She also had mild lower extremity and abdominal wall pitting edema. Heart sounds were not muffled and organomegaly was difficult to appreciate due to obese body habitus. Her clinical presentation was concerning for appendicitis; hence, computed tomography (CT) of the abdomen and pelvis was ordered along with laboratory studies including liver function panel.

**Image 1:**
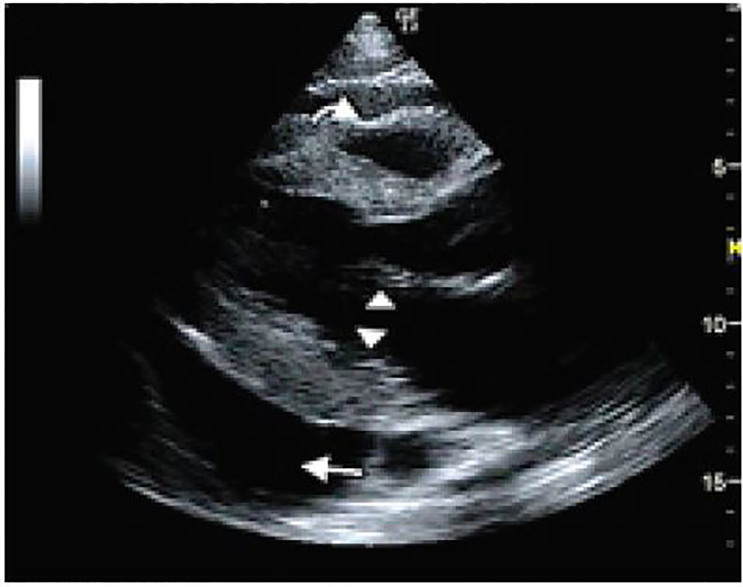
Parasternal long-axis view demonstrating a pericardial effusion (straight arrow) with right ventricular collapse (curved arrow) during diastole, as evidenced by opening of the mitral valves (arrowheads)

**Image 2:**
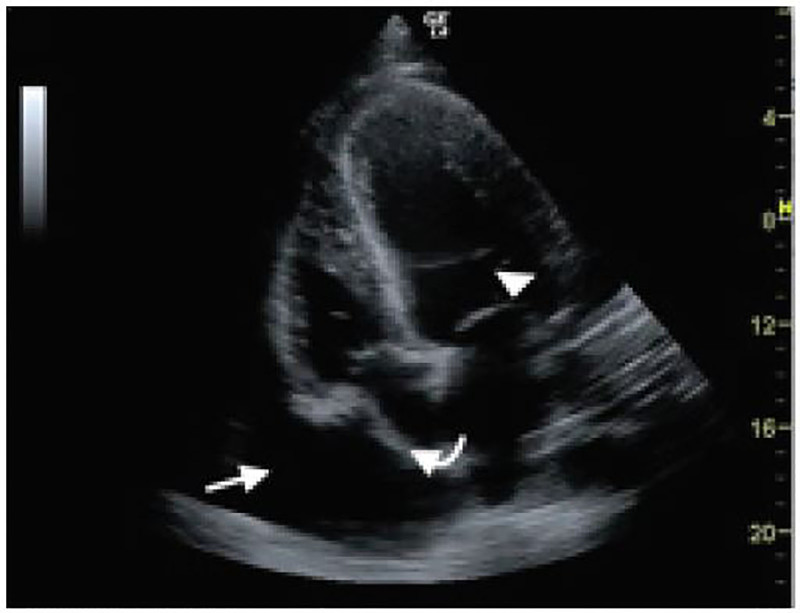
Apical four-chamber view illustrating a pericardial effusion (straight arrow) along with right atrial collapse (curved arrow) during ventricular systole (Note the mitral valve [arrowhead] is closed.)

**Image 3:**
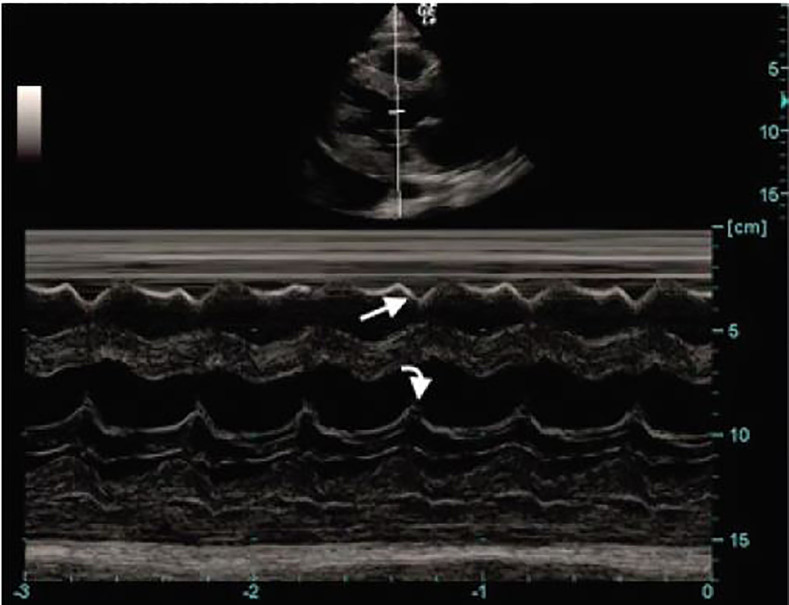
M-mode tracing demonstrating collapse of the right ventricle free wall (straight arrow) during the diastolic phase (mitral valve opening) (curved arrow).

Her laboratory analysis was significant for an elevated creatinine of 1.54 mg/dL (0.6–1.1 mg/dL). Her hemoglobin was 11.4 g/dL (12–15 g/dL) with microcytosis. The remaining laboratory studies including a liver function panel were within normal limits. Abdominal CT showed hepatomegaly and a large pericardial effusion. An ECG was immediately performed, which did not show any evidence of electrical alternans or a low voltage QRS. A point-of-care echocardiogram was performed, which showed a large pericardial effusion with early diastolic collapse of the right ventricular (RV) free wall as well as right atrial collapse during ventricular systole (Images 1, 2). An M-mode tracing was used to further ascertain the RV free wall collapse during the diastolic phase (evidenced by mitral valve opening) (Image 3). This indicated that intrapericardial pressure transiently exceeded the RV pressure, resulting in early tamponade physiology.

Cardiothoracic surgery was consulted for definitive management. During her stay in the ED the patient remained tachycardic but her blood pressure decreased to 140/110 mmHg, also indicative of tamponade physiology due to the narrow pulse pressure. The patient was taken to the operating room for pericardial window. A total of 1,100 mL of serous fluid was removed from the pericardium with immediate improvement of her hemodynamic state.

**CPC-EM Capsule**

What do we already know about this clinical entity?

Lupus is a commonly encountered disease process that can affect multiple organ systems. Pericarditis is a well-known manifestation.

What makes this presentation of disease reportable?

Undiagnosed lupus can present with a slow developing pericardial effusion, leading to atypical symptoms of pericardial tamponade.

What is the major learning point?

Point-of-care ultrasonography can provide critical information in patients with vital sign abnormalities, and cardiac tamponade can present as abdominal pain.

How might this improve emergency medicine practice?

More vigilant utilization of point-of-care ultrasonography can lead to quicker diagnoses and thus better patient outcomes.

Post-operatively the patient underwent laboratory analysis to assess for SLE. She was found to be antinuclear antibody positive. Extractable nuclear antigen screen was also positive with elevated anti-Smith antibody, anti-ribonucleoprotein antibody, anti-Sjogrens syndrome-related type A antibody, and anti-cardiolipin antibody, confirming her diagnosis of SLE. Patient was managed with oral steroids and discharged in stable condition with outpatient rheumatology follow-up.

## DISCUSSION

SLE is an autoimmune disease process with a wide variety of initial presentations. It is commonly known as the “great imitator” as it can pathologically affect any organ system.^1^ Patients classically have integumentary and renal involvement with lupus with an additional concordance with prothrombotic events in the presence of anti-phospholipid antibodies. Lupus pericarditis with secondary cardiac tamponade is much less common as a presenting symptom. Many case reports have documented this as a presenting symptom; however, most of those patients present with a current or recent history of chest pain or dyspnea.^4^,^5^,^6^,^7^,^8^,^9^ Abdominal pain from hepatic congestion, as seen in this patient, has only been reported in two case studies reported since 1995.^3^

Although our patient was complaining of abdominal discomfort, on presentation she was persistently tachycardic. She also had mild pitting edema of her lower extremities and abdominal wall. These findings, although subtle, were significant enough to warrant point-of-care echocardiographic assessment for global cardiac function and the presence of a pericardial effusion.

## CONCLUSION

The etiology of a pericardial effusion can vary greatly, including post-myocardial infarction, infectious, autoimmune, uremic, malignant or structural. The rate of accumulation of the effusion determines the symptomatology of the patient. Among the effusions that progress to tamponade, patients are known to classically exhibit Beck’s triad (hypotension, jugular venous distension and muffled heart sounds). In patients who develop subacute pericardial tamponade, the characteristic profile may not be apparent. In the present case, the patient was hypertensive and tachycardic but heart sounds were not muffled. With regard to atypical symptoms, it is prudent to remain vigilant of abnormalities in vital signs in light of symptomatic propriety given her tachycardia and peripheral edema without any known underlying cause. Delay in pericardial evacuation can be life-threatening. This case re-establishes the importance of using point-of-care ultrasonography as an adjunct to physical examination to help improve time to definitive therapy and disposition.
